# Sleep habits are associated with cognition decline in physically robust, but not in frail participants: a longitudinal observational study

**DOI:** 10.1038/s41598-022-15915-y

**Published:** 2022-07-08

**Authors:** Shu-Chun Chuang, I.-Chien Wu, Jen-Jen Chang, Yi-Fen Tsai, Chiu-Wen Cheng, Yen-Feng Chiu, Hsing-Yi Chang, Marion M. Lee, Chih-Cheng Hsu, Chao Agnes Hsiung

**Affiliations:** 1 Institute of Population Health Sciences, National Health Research Institutes, Zhunan, Miaoli Taiwan; 2 Department of Epidemiology and Biostatistics, College for Public Health and Social Justice, Saint Louis University, St. Louis, MO USA; 3 Department of Epidemiology and Biostatistics, University of California San Francisco, San Francisco, CA USA

**Keywords:** Gastroenterology, Risk factors

## Abstract

Frail older adults are vulnerable to stressors; thus, sleep related cognition impairment might more greatly affect frail than healthy older adults. In the present study, we investigated whether the association between sleep problems and cognition varies with physical frailty status (modified from Fried et al.). Participants 55 years and older who completed a baseline and follow-up questionnaire (median follow-up: 5.5 years), were included in the analysis. Sleep parameters were evaluated in an interview at the baseline. Cognitive decline was defined as a loss of 3 or more points on the Mini-Mental State Examination (MMSE) at follow-up. Associations between sleep problems and cognitive decline were examined using logistic regression and were stratified by baseline physical frailty status, adjusted for potential confounders. A short total sleep duration (< 5 vs. 7–9 h, odds ratio (OR) = 1.88, 95% confidence interval (CI) 1.18–3.00), excessive daytime sleepiness (OR = 1.49, 95% CI 1.04–2.13), low sleep efficiency (< 65% vs. ≥ 85%, OR = 1.62, 95% CI 1.07–2.46), and insomnia complaints (OR = 2.34, 95% CI 1.23–4.43) were associated with MMSE decline in physically robust. The association was stronger for the sleep summary score, which summarized abnormal sleep duration, excessive daytime sleepiness, and insomnia complaints ($\ge$ 2 vs. 0, OR = 3.79, 95% CI 2.10–6.85, p < 0.0001). Due to the low prevalence of frailty in this community-dwelling population, the statistical power to detect an association was low. More evidence is needed to clarify the role of sleep in the progression of cognitive decline in frail individuals.

## Introduction

Sleep is essential for normal brain functioning, maintaining the stability of neuropsychological reactions for attention, arousal, and vigilance^1,2^. Longitudinal studies showed that excessive daytime sleepiness increases amyloid-β concentrations^3^ and that shorter sleep duration and poor sleep quality are associated with an increased amyloid-β burden in community-dwelling older adults^4,5^. Previous studies in Taiwan indicated that, based on a nationwide survey, the prevalence of insomnia was 44.7% in men and 62.5% in women^6^; however, the prevalence in National Health Insurance claim data was only 5.5% in men and 8.6% in women^7^. That discrepancy might arise if people who self-report insomnia are not found to meet the diagnostic criteria for insomnia or if only a few people complaining of insomnia have sought medical help.

As some aspects of frailty might potentially be reversible, identifying individuals at high risk for dementia (such as those with frailty) and developing upstream interventions might prevent progression to dementia and promote a healthy lifespan^8^. One published study showed that poor sleep mediated the relationship between frailty and cognitive functioning^9^. On the other hand, sleep problems such as insomnia, insufficient sleep, or a disrupted circadian rhythm, can result in frailty and speed cognition decline. A study conducted by the Mayo Clinic indicated that excessive daytime sleepiness and fatigue are associated with accelerated brain aging in participants 50 years and older^10^. On the other hand, because frail older adults are more vulnerable to stressors, recovery from sleep-related cognition loss might be difficult for people with frailty^11,12^. Thus, we hypothesized that the impact of sleep problems on cognition might be different depending on physical frailty status.

The Healthy Ageing Longitudinal Study in Taiwan (HALST), a prospective follow-up cohort study of community-dwelling older adults, provides an opportunity to observe physical and cognitive changes in later life. The goal of the study was to investigate the association between sleep problems and cognitive decline in a group of non-clinical community-dwelling older adults in Taiwan over a 5-year follow-up. For present work, we particularly wanted to know whether that association differed by physical frailty status.

## Results

Table 1 presents the baseline characteristics of the participants. Participants with frailty were more likely to be older, to have a lower degree of education, to have more chronic diseases, and to score lower in social network assessment and higher on the Center for Epidemiologic Studies–Depression (CES-D) instrument. The mean change in the score on the Mini-Mental State Examination (MMSE) over the 5-year follow-up was − 1.32 (SD = 2.53) for participants who were robust, − 1.86 (SD = 3.29) for those with pre-frailty, and − 2.97 (SD = 4.06) for participants with frailty.

**Table 1:** Baseline characteristics of the study population.

Characteristics at baseline	Total ^a^		Robust		Pre-frail		Frail		*p*-value
	N	%	N	%	N	%	N	%	
Age (mean ± SD)	68.3 ± 7.7		66.6 ± 7.0		70.2 ± 7.8		73.5 ± 7.7		< 0.001
**Sex**									
Men	1030	47.3	599	48.2	391	48.5	24	32.4	0.027
Women	1147	52.7	644	51.8	415	51.5	50	67.6	
**Education**									
Illiteracy	244	11.2	76	6.1	127	15.8	24	32.4	< 0.001
Primary school	1013	46.5	557	44.8	392	48.6	38	51.4	
More than primary school	920	42.3	610	49.1	287	35.6	12	16.2	
**Current working status**									
No	1536	70.6	844	67.9	578	71.7	71	95.9	< 0.001
Yes	641	29.4	399	32.1	228	28.3	3	4.1	
**Smoking**									
Never	1562	71.8	920	74.0	546	67.7	57	77.0	0.024
Former	345	15.8	183	14.7	142	17.6	11	14.9	
Current	270	12.4	140	11.3	118	14.6	6	8.1	
**Drinking**									
Never	1357	62.3	750	60.3	514	63.8	52	70.3	< 0.001
Former	187	8.6	83	6.7	90	11.2	9	12.2	
Current	633	29.1	410	33.0	202	25.1	13	17.6	
**Betel nut chewing**									
Never	1934	88.8	1116	89.8	699	86.7	69	93.2	0.074
Former	180	8.3	89	7.2	82	10.2	5	6.8	
Current	63	2.9	38	3.1	25	3.1	0	0.0	
**Exercise**									
No	320	14.7	0	0.0	253	31.4	55	74.3	< 0.001
Some	1528	70.2	1007	81.0	469	58.2	17	23.0	
Meet the recommendation ^b^	329	15.1	236	19.0	84	10.4	2	2.7	
**Chronic diseases**									
0–2	969	44.5	633	50.9	294	36.5	19	25.7	< 0.001
3–5	936	43.0	502	40.4	373	46.3	40	54.1	
≥ 6	272	12.5	108	8.7	139	17.3	15	20.3	
**Social network score**									
≥ 8	1231	56.6	765	61.5	416	51.6	22	29.7	< 0.001
6–7	577	26.5	314	25.3	233	28.9	21	28.4	
0–5	369	17.0	164	13.2	157	19.5	31	41.9	
**CESD**									
< 16	2075	95.3	1226	98.6	749	92.9	53	71.6	< 0.001
≥ 16	102	4.7	17	1.4	57	7.1	21	28.4	
**MMSE score (mean ± SD)**	26.53 ± 3.58		27.38 ± 2.83		25.76 ± 3.89		23.41 ± 4.54		< 0.001
MMSE score < 24	301	13.8	96	7.7	148	18.4	35	47.3	< 0.001

*CES-D* center for epidemiologic studies-depression scale, *MMSE* mini-mental status examination, *N* population size, *SD* standard deviation.

^a^A total of 54 participants (2.5%) were missing physical frailty status.

^b^Moderate exercise for 150 min per week or vigorous exercise for 75 min per week.

Table 2 presents the baseline sleep parameters for the study population. Briefly, 10.9% of the participants slept for less than 5 h, and 7.7% for longer than 9 h, Insomnia complains during the preceding month of the interview were expressed by 5.1% of the participants and 8.6% of the participants had been told by a doctor that they had insomnia. Those proportions were all higher in participants with frailty.

**Table 2:** Baseline sleep parameters for the study population.

	Total ^a^		Robust		Prefrailty		Frailty		*p*-value
	N	%	N	%	N	%	N	%	
**Midpoint of sleep**									
23:00–01:00	264	12.1	127	10.2	109	13.5	16	21.6	0.010
01:00–03:00	1515	69.7	892	71.7	544	67.6	46	61.2	
03:00–05:00	345	15.9	201	16.2	128	15.9	9	12.2	
05:00–23:00	51	2.3	23	1.9	24	3.0	3	4.1	
Missing	2		0		1		0		
**Total sleep duration (h)**									
< 5	237	10.9	111	8.9	102	12.7	15	20.6	< 0.001
5–7	902	41.6	537	43.2	323	40.3	23	31.5	
7–9	864	39.8	520	41.9	308	38.5	17	23.3	
≥ 9	166	7.7	74	6.0	68	8.5	18	24.7	
Missing	8		1		5		1		
**Nap**									
No	926	42.5	525	42.2	343	42.6	32	43.2	0.979
Yes	1251	57.5	718	57.8	463	57.4	42	56.8	
**Excessive daytime sleepiness**									
≤ 10	1708	84.4	1013	85.1	606	83.7	51	77.3	0.194
> 10	316	15.6	177	14.9	118	16.3	15	22.7	
Missing	153		53		82		8		
**Sleep efficiency**									
≥ 85%	1141	52.7	706	56.8	391	48.9	23	31.5	< 0.001
75–85%	457	21.1	266	21.4	163	20.4	17	23.3	
65–75%	249	11.5	135	10.9	96	12.0	11	15.1	
< 65%	320	14.8	135	10.9	150	18.8	22	30.1	
Missing	10		1		6		1		
Insomnia complaint	110	5.1	47	3.8	51	6.3	8	10.8	0.002
Insomnia diagnosis	188	8.6	79	6.4	93	11.5	10	13.5	< 0.001
Self-reported sleep or sedative drugs	325	14.9	153	12.3	145	18.0	17	23.0	0.003

^a^Physical frailty status was missing for 54 participants (2.5%).

**Figure 1:**
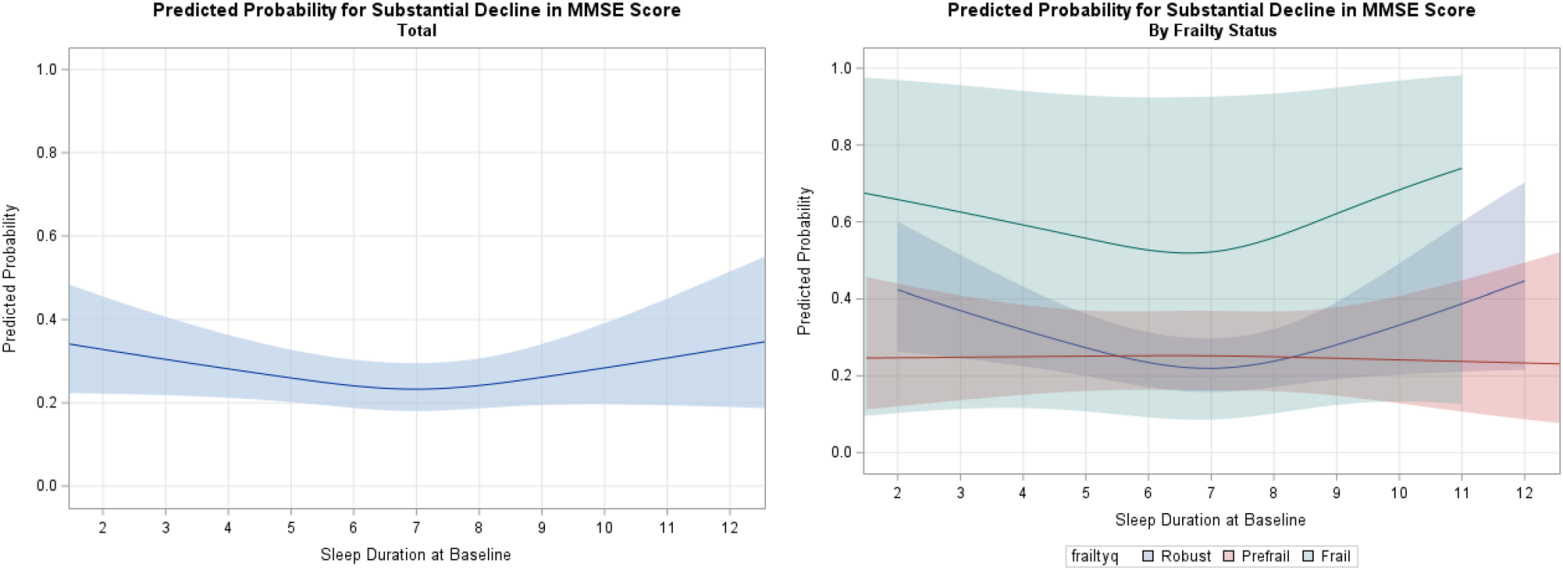
Restricted cubic spline plots of predicted probability of MMSE decline for total sleep duration. *MMSE* mini-mental status examination.

Table 3 shows the association between a declining score on the MMSE and baseline sleep parameters. Overall, only short sleep duration was associated with a declining score on the MMSE (< 5 vs. 7 ~ 9 h, odds ratio (OR) = 1.41, 95% confidence interval (CI) 1.00–1.98). When participants were stratified by physical frailty status, short sleep duration (OR = 1.88, 95% CI 1.18–3.00), excessive daytime sleepiness (OR = 1.49, 95% CI 1.04–2.13), low sleep efficiency (< 65% vs. $\ge$ 85%, OR = 1.62, 95% CI 1.07–2.46) and insomnia complaints (OR = 2.34, 95% CI 1.23–4.43) were associated with a declining score on the MMSE in the participants who were robust, and the association was stronger for the sleep summary score ($\ge$ 2 vs. 0, OR = 3.79, 95% CI 2.10–6.85, p < 0.0001). In participants with pre-frailty, diagnosed insomnia was associated with lower odds of a declining score on the MMSE (OR = 0.55, 95% CI 0.30–0.97). No statistically significant association was observed in participants with frailty. When sleep duration was expressed as continuous variable, the probability of a declining score on the MMSE was lowest at approximately 7 h for participants who were robust and for those with frailty alike, but was flat for participants with pre-frailty (Fig. 1).

**Table 3:** Association between baseline sleep parameters and decline in the score on the Min-Mental Status Examination^a^ based on physical frailty status.

	Total					Robust				Prefrail				Frail				*P-heterogeneity*
	Diff > -3	Diff ≤ -3	OR^b^	95% CI		Diff > -3	Diff ≤ -3	OR^b^	95% CI	Diff > -3	Diff ≤ -3	OR^b^	95% CI	Diff > -3	Diff ≤ -3	OR^b^	95% CI	
**Midpoint of sleep**																		
23:00–01:00	157	96	1.04	(0.77, 1.41)		79	44	1.22	(0.80, 1.86)	62	43	0.86	(0.53, 1.36)	8	6	0.82	(0.23, 2.82)	0.861
01:00–03:00	1032	460	1.00			646	238	1.00		346	187	1.00		21	24	1.00		
03:00–05:00	262	75	0.86	(0.64, 1.17)		163	36	0.77	(0.51, 1.16)	87	36	1.15	(0.71, 1.85)	6	2	0.27	(0.03, 1.49)	
05:00–23:00	39	10	0.67	(0.31, 1.45)		17	4	0.70	(0.23, 2.20)	19	5	1.08	(0.35, 2.90)	3	0	–	–	
**Total sleep duration (h)**																		
< 5	147	82	1.41	(1.00, 1.98)		71	39	1.88	(1.18, 3.00)	62	36	1.02	(0.60, 1.74)	8	7	1.05	(0.25, 4.40)	0.626
5–7	638	251	1.01	(0.81, 1.26)		398	133	1.06	(0.79, 1.42)	212	105	1.00	(0.69, 1.44)	15	7	0.52	(0.13, 2.03)	
7–9	606	241	1.00			391	125	1.00		197	102	1.00		7	9	1.00		
≥ 9	96	63	1.28	(0.87, 1.88)		45	25	1.45	(0.83, 2.53)	40	27	1.06	(0.59, 1.90)	8	9	1.40	(0.34, 5.95)	
**Nap**																		
No	659	250	1.00			393	127	1.00		230	104	1.00		18	13	1.00		0.900
Yes	832	391	1.13	(0.92, 1.38)		512	195	1.13	(0.86, 1.48)	285	167	1.15	(0.83, 1.59)	20	19	1.04	(0.38, 2.77)	
**Excessive daytime sleepiness**																		
≤ 10	1198	476	1.00			749	249	1.00		396	197	1.00		26	22	1.00		0.535
> 10	200	109	1.26	(0.95, 1.66)		117	60	1.49	(1.04, 2.13)	75	38	1.01	(0.62, 1.61)	7	7	1.46	(0.43, 5.07)	
**Sleep efficiency**																		
≥ 85%	793	329	1.00			520	179	1.00		248	132	1.00		10	12	1.00		0.628
75%-85%	323	127	0.90	(0.69, 1.17)		203	59	0.84	(0.59, 1.19)	105	56	0.95	(0.62, 1.45)	10	7	0.66	(0.14, 2.94)	
65%-75%	176	70	0.94	(0.67, 1.30)		99	35	1.02	(0.65, 1.58)	65	30	0.82	(0.48, 1.37)	6	5	0.84	(0.18, 3.92)	
< 65%	194	113	1.21	(0.90, 1.63)		83	49	1.62	(1.07, 2.46)	92	52	0.97	(0.61, 1.51)	12	8	0.65	(0.15, 2.58)	
**Insomnia complaint**																		
No	1423	602	1.00			878	302	1.00		480	257	1.00		35	28	1.00		0.022
Yes	68	39	1.38	(0.88, 2.18)		27	20	2.34	(1.23, 4.43)	35	14	0.71	(0.34, 1.41)	3	4	2.76	(0.56, 15.4)	
**Insomnia diagnosis**																		
No	1358	590	1.00			851	297	1.00		444	251	1.00		33	28	1.00		0.021
Yes	133	51	0.94	(0.64, 1.37)		54	25	1.52	(0.89, 2.58)	71	30	0.55	(0.30, 0.97)	5	4	1.12	(0.22, 5.95)	
**Self-reported hypnotic or sedative drug use**																		
No	1255	557	1.00			788	286	1.00		411	232	1.00		28	25	1.00		0.644
Yes	236	84	0.78	(0.58, 1.06)		117	36	0.81	(0.53, 1.25)	104	39	0.72	(0.45, 1.12)	10	7	0.99	(0.27, 3.57)	
**Sleep summary score**^c^																		
0	993	368	1.00			647	199	1.00		311	153	1.00		14	11	1.00		0.131
1	343	163	1.10	(0.86,	1.40)	193	80	1.31	(0.95, 1.80)	128	65	0.87	(0.59, 1.28)	17	13	1.31	(0.43, 4.16)	
≥ 2	58	52	2.54	(1.64, 3.95)^d^		26	30	3.79	(2.10, 6.85) ^d^	28	16	1.37	(0.65, 2.87)	2	5	4.43	(0.73, 33.6)	

*CES-D* center for epidemiologic studies–depression scale, *CI* confidence interval, *h* hours, *OR* odds ratio, *NE* not estimable.

^a^A substantial decline in score was defined as a drop of 3 or more points at the follow-up assessment.

^b^Odds ratios were adjusted for age at the baseline assessment, sex, center, education, current working status, betel nut chewing status, number of chronic diseases, social network score, and CES-D score (and physical frailty status for total).

^c^Sleep summary score was created for having short or long sleep duration (< 5 or ≥ 9 h), excessive daytime sleepiness, and insomnia complaints.

^d^*P* < 0.005.

## Discussion

Our study indicates that the abnormal sleep duration, excessive daytime sleepiness, low sleep efficiency, and insomnia complaints are associated with a declining score on the MMSE in participants who were physically robust over a 5-year follow-up period. However, no association was observed in participants with frailty, even when combining those with prefrailty and frailty. Heterogeneity for the associations of insomnia and declining score on the MMSE across frailty status were detected (Supplementary Table S4). Sensitivity analyses restricting data to those with a normal score on the MMSE ($\ge$ 24) at baseline or redefining a score on the MMSE as a drop greater than or equal to 4 points produced similar results (Supplementary Table S2).

In general, our study agrees with previous studies and demonstrated a U-shaped association between total sleep duration and declining score on the MMSE^13^ and between insomnia and a declining score on the MMSE^14^ in participants who were physically robust (Table 3 and Fig. 1). However, we were not able to observe an association in individuals with frailty. That observation seemed contrary to our original hypothesis. Nevertheless, frailty is a heterogeneous entity that manifests as advanced aging and dysregulated homeostasis, and that could arise from other causes^23,24^. Sleep-related cognition loss might be only a subtype. However, we did not observe associations between sleep problems and cognition decline in any of the subtype of physical frailty proposed by Liu et al^24^ and Huang et al^23^ (data not shown).

Another probable reason could be that these participants with frailty were already experiencing sleep-related cognition loss, as demonstrated in Tables 1 and 2, that they had higher prevalence of sleep problems and lower MMSE scores at baseline. Depending on the onset of frailty, a declining MMSE score might present earlier in the individual’s life course. Restricting the analysis to individuals who had a normal MMSE score at baseline revealed stronger, but not statistically significant, results (Supplementary Table S2). Another explanation could be that the variance in MMSE scores was larger in the group with frailty than in the robust group (Table 1), thus limiting the possibility of observing a significant association between insomnia and a declining score on the MMSE. Finally, our study might be underpowered to detect an association between sleep problems and cognition decline in participants with frailty. Assuming an exposure prevalence of 40% in the population with frailty, 150 participants would be needed to have 80% power to detect a risk ratio of 2. The non-association in this subgroup could also be a result of false negative due to its limited sample size.

The effects of treatments for insomnia on preventing or delaying cognitive impairment have been inconsistent. A Cochrane review^26^ found no cognitive benefits of drug treatments for insomnia in patients with dementia. Another study^27^ that used behavioral treatment in a group of cognitively intact patients with insomnia also failed to show any benefits for cognitive function. Similarly, we observed no risk or benefit for sleep and sedative drug use in our study. Although 8.6% of the participants in our cohort self-reported that they were diagnosed with insomnia, 14.8% reported that they had taken sleep or sedative drugs regularly during the preceding month. We were able to confirm those self-reports from the drug bags in 60.7% of the participants. Notably, however, some of those drugs might have been prescribed for psychiatric conditions, rather than insomnia, potentially explaining the non-association in our study.

As people age, their sleep patterns change. For example, with weakening of circadian signals during aging, earlier sleep and waking tend to be more common in older adults than in a younger population^28^. Some older adults might present a disrupted circadian rhythm^29^. Sleep problems are well documented to predict risk for frailty and are prevalent in participants with frailty^8,30^. Disruptions of circadian rhythms and sleep could be a precursor or a driver of neurodegenerative diseases^31^. In our study, participants with frailty, compared with participants who were robust and pre-frailty, presented an earlier or abnormal midpoint of sleep. Circadian disruption might not be the only factor to increase the prevalence of frailty; frailty might also change sleep habits. However, no association was observed between the midpoint of sleep and cognitive decline in participants who were robust or who had pre-frailty, and frailty. A reverse causation, that is, cognitive decline was already present in participants with frailty; thus our ability to detect an association between midpoint of sleep and further decline was limited.

Taken together, accumulated damage to the brain during aging because of cerebral ischemia, head trauma, toxins, and excess stress hormones could be producing age-related cognitive impairments^32^. Sleep deprivation is known to result in free radicals and neurotoxic waste accumulation in the brain, which is associated with sleep-related cognition loss^18,19^. Our observations suggest that, although sleep-related cognition loss might affect healthy older adults, sleep problems likely play a minor role or are an outcome of aging-related deteriorations. However, given the observational nature of our study and its small sample size of the population with frailty, we were not able to confirm a causal association. More research with larger frail sample is needed to confirm our observation.

Beyond the small sample of the population with frailty, the major limitation of our study was the self-reported sleep measurement. Recall bias and lack of accuracy in the responses might have occurred. Nevertheless, use of a sleep questionnaire is an easier, more economical, more acceptable, and more practical approach to measuring sleep patterns in a population setting with a low participation burden. Moreover, recall bias is likely nondifferential by outcome status, which would bias the point estimate toward the null value. Secondly, we used the MMSE as the only instrument to measure cognition. The MMSE is known to be insufficiently sensitive to detect a mild decline in cognition and could be confounded by culture and language. We aimed to reduce the confounding effect from language by interviewing all participants in their primary language, including Taiwanese and Hakka, in addition to Mandarin. Thirdly, although we excluded participants who self-reported a dementia diagnosis, we cannot preclude the possibility that people with cognitive decline were included in our analysis. However, when we restricted our analyses to participants whose MMSE was greater than 24, results were similar (supplementary Table S2). Fourthly, almost 30% of the participants had died or dopped out at follow-up. However, including these participants in the analysis as separate outcomes in a nominal logistic regression did not change the results dramatically (Supplementary Table S3). Furthermore, half of the cohort had not been scheduled for the follow-up interview yet when the analysis was done. These participants had higher education and higher MMSE at baseline. The current study might be not be able generalized to those with higher education. Finally, although ORs usually overestimate relative risks when the outcomes are common in high-risk populations, (for example, those with frailty) is the estimates are usually considered appropriate, especially when the range of underlying risks in the populations is large^33^.

Our study also has several strengths. Its prospective nature provided a unique opportunity to observe change in the cognitive function of older adults. All participants in our cohort were followed using the same protocol during the follow-up assessment, thus reducing detection bias. Moreover, our cohort included a relatively large group of cognitively intact, community-dwelling participants who were able to supply detailed data about other covariates, such as education, lifestyle, comorbidities, medications, depression, and social network, thereby enabling us to reduce the residual confounding and test the independent association of sleep parameters.

In summary, our results indicated that abnormal sleep duration, excessive daytime sleepiness, low sleep efficiency, and insomnia complaints were consistently associated with cognitive decline in participants who were physically robust. Due to the low prevalence of frailty in this community-dwelling population, the statistical power to detect an association was low. More evidence is needed to clarify the role of sleep in the progression of cognitive decline in older adults with frailty.

## Methods

### The healthy ageing longitudinal study in Taiwan (HALST)

The HALST included 5,664 participants across Taiwan starting in 2008. The cohort has previously been described^34^. In brief, all residents (55 years of age or older) living within the catchment area of seven collaborating hospitals were eligible for the study. Participants were excluded if they had highly contagious infectious diseases (e.g. scabies, open pulmonary tuberculosis), diagnosed dementia, severe illness (e.g. cancer under treatment) or were bed-ridden (unable to move), severe mental disorder (cannot be communicated with), mutism, hearing impairment, or blindness (unable to complete the interview), and other conditions such as living in a long-term care facility or being hospitalized. Interviewers were trained to conduct the face-to-face interview using the participant’s primary language and to take physical performance measurements strictly following the study protocols. Participants whose baseline MMSE score was less than 16 were excluded from the HALST.

All participants who completed the home visit at baseline were re-assessed in 2013. By the time of the present analysis, 3,120 participants had completed the follow-up; the research team was still contacting or making appointments with the remaining 2544 participants. For the present study, 7 of the 3120 participants who had complete follow-up were excluded because of self-reported diagnosed dementia at baseline. Another 320 had died, 103 were too ill to participate, 399 refused participation, and 114 could not be contacted, leaving 2177 participants (69.9%) to complete the assessment. In general, compared with the participants who dropped out, the participants included in our study were younger, had a higher education, greater social network scores, and less depression (Supplementary Table S1). The median time from baseline to follow-up was 5.5 years (range 4.5–7.2 years).

Written informed consent forms were completed by every participant at baseline and at follow-up. The study was approved by the Institutional Review Board at the National Health Research Institutes and the collaborating hospitals. All procedures were performed in accordance with the relevant guidelines and regulations.

### Sleep parameters

The sleep questionnaire used here was also used in the National Health Interview Survey in Taiwan. During the baseline assessments, all participants were asked questions pertaining to their sleep during weekdays. The midpoint of sleep was calculated as the halfway point between the time the participant went to bed and woke up. Sleep duration was the sum of self-reported total time in bed per 24 h including nocturnal sleep and nap during the day. Sleep efficiency was defined as the number of hours of self-reported sleep divided by the hours in bed at night. Insomnia complaints were measured using these three questions: during the past month, did you have trouble falling asleep? Did you wake up during the night and have trouble getting back to sleep? Did you wake up too early in the morning and be unable to get back to sleep? The questions were scored using a 5-point Likert-type scale (1 = never, 2 = seldom (1 time or less), 3 = sometimes (2–4 times), 4 = usually (5–15 times), and 5 = all the time (16–30 times). A response of 4 or 5 was considered positive for any given insomnia complaint. Participants with insomnia complaints were defined as those who were having trouble falling asleep, staying asleep, and waking up too early^35^. The participants were also asked whether they had ever received a diagnosis of insomnia by a doctor. The Epworth Sleepiness Scale was used to assess excessive daytime sleepiness^36^. The use of sleep or sedative drugs was self-reported. Because sleep problem has multifactorial features and they are closely related to one another, a summary score based on preliminary results that combined abnormal duration (< 5 or $\ge$ 9 h), excessive daytime sleepiness (Epworth Sleepiness Scale > 10), and insomnia complaints (“yes”) was created to reflect the combined effects.

### Physical frailty

The definition of physical frailty was modified from Fried et al.^25^. “Weight loss” was defined as a self-reported involuntary loss of more than 4.5 kg of body weight during the preceding year. “Exhaustion” was defined by a self-response of “a moderate amount of the time” or “most of the time” to either of the statements “I felt that everything I did was an effort” or “I could not get going” on the CES-D. Participants were asked about the type, frequency, duration, and intensity of exercise and work-related physical activities in which they engaged over the preceding year. “Low physical activity” was defined as the lower 20% sex-specific physical activity (kilocalories/week) in the total study population (N = 5664). The faster of two walks was used to define the walking speed in the 3- or 4-m walking test. “Slow walking speed” was defined as the lower 20% sex- and height-specific cutoffs. Handgrip strength was measured using a North Coast hand dynamometer (North Coast Medical Inc., Gilroy, CA, USA). The best performance (in kilograms) in three trials of the dominant hand was used for the analysis. Weakness” was defined as the lower 20% of the sex- and body-mass-index-specific cutoffs in the handgrip strength test. All cutoffs and the prevalence of frailty at baseline in our study are presented in Table 4. Following the definition in Fried et al.^25^, participants without any of these foregoing conditions were considered to be either physically robust, or to have pre-frailty if they had one or two conditions, and frailty if they had three or more conditions.

**Table 4:** Definition for physical frailty.

Characteristics	Definition		%
Weight loss	Lost > 4.5 kg in preceding year		2.7
Exhaustion	Agreement with either or both statements on the CES-D		5.4
Low physical activity	The lowest sex-specific 20% of study population (M: < 105.154 kcal/week, F: < 46.08 kcal/week)		16.5
Slowness	On the 3 or 4-m walking test (the lowest sex-specific 20% of study population)		18.2
	**Men**		
	Height ≤ 165 cm	≤ 0.63 m/s	
	Height > 165 cm	≤ 0.74 m/s	
	**Women**		
	Height ≤ 153.2 cm	≤ 0.54 m/s	
	Height > 153.2 cm	≤ 0.65 m/s	
Weakness	Handgrip strength (the lowest sex-specific 20% of study population)		16.9
	**Men**		
	BMI ≤ 22.3	≤ 25.3	
	BMI 22.4–24.4	≤ 28	
	BMI 24.5–26.5	≤ 27.7	
	BMI > 26.5	≤ 29.2	
	**Women**		
	BMI ≤ 22	≤ 16	
	BMI 22.1–24.2	≤ 16.7	
	BMI 24.3–26.8	≤ 16.7	
	BMI > 26.8	≤ 15.8	
Frailty	Robust		58.5
	Pre-frailty		38.0
	Frailty		3.5

### MMSE decline

Global cognitive function was assessed using the MMSE questionnaire at baseline and at follow-up. The MMSE score range from 0 to 30 and is commonly used in primary care and research settings with older adults. The score has been shown to be sensitive in detecting moderate-to-severe cognitive impairment^37^. The mean drop from the baseline to follow-up assessment in participants with frailty was 2.97; thus, we arbitrarily choose a drop of 3 or more points to be a substantial drop in cognition. Poor cognition was defined as an MMSE score below 24^38^. Given the effects of education and incomplete results, a lower cutoff was set separately for individuals with incompletion and illiteracy^38^.

### Other covariates

A number of covariates were selected as potential confounders based on their known associations in the literature with sleep and cognition, including age; sex; education level (illiteracy, primary school, or more than primary school); smoking; drinking; and betel nut chewing status (never, former, or current); personal history of medical conditions (including diabetes, heart disease, stroke, hyperlipidemia, asthma, chronic respiratory tract disease, cancer, gastric disease, liver and gallbladder disease, cataract, gout, anemia, kidney diseases, arthritis, spurs, osteoporosis, fracture, and mental illness); social network score and CES-D scores (< 16 or $\ge$ 16). Given the varying urbanization status of the study centers, study center was included as one of the covariates. Initially, we included smoking, drinking, and betel nut chewing status in one model, but because only betel nut chewing status remained statistically significant, only betel nut chewing status was included in the final model. The social network score was calculated on the basis of six questions about the participant’s interactions with immediate family members, relatives, friends, and neighbors, and about participation in community activities. Each question was scored from 0 to 2, with a higher score indicating higher frequency of interaction or participation. A social network score was the summary score of the six questions (range: 0–12).

### Statistical analysis

The baseline characteristics of the participants were assigned to categories and compared using the chi-squared test. Midpoint of sleep, total sleep duration^39^, sleep efficiency^40^, and excessive daytime sleepiness^36^, were categorized based on the published literatures. The associations between MMSE decline and baseline sleep parameters were analyzed using multivariate logistic regression models. Sleep parameters were tested in separate models to avoid multicollinearity.

The linear association between the continuous measures of total sleep duration and MMSE decline was examined using restricted cubic spline models with knots specified at 5, 7, and 9 h. A drop of 4 or more points in MMSE score was selected for a sensitivity analysis, as well as for baseline participants with an MMSE-normal score (Supplementary Table S2). Because a substantial number of participants had died, were too ill to participate, had dropped out, or were lost to follow-up at the follow-up assessment, which might have led to selective survival bias, we treated such events as separate outcomes and analyzed them using nominal logistic regressions (Supplementary Table S3). Given the small sample of the population with frailty, participants who were classified as pre-frailty and frailty were grouped to determine whether the effects might or might not emerge from the analyses (Supplementary Table S4).

All the analyses were performed on the total sample stratified by baseline physical frailty status and adjusted for the covariates described earlier. We assessed heterogeneity across frailty status by the likelihood ratio test. Because sample sizes after stratification were small, Firth logistic regression was used^41^. The significance level was set at a *p*-value less than 0.05. Analyses were performed using SAS statistical software application (SAS Institute, Cary, NC, USA).

### Ethics approval and consent to participant

Informed consent forms were completed by each participant in written at baseline and at follow-up. The study was approved by the Institutional Review Board at the National Health Research Institutes and the collaborative hospitals. All methods were performed in accordance with the relevant guidelines and regulations.

### Consent to participate/consent to publish

Written informed consents were obtained from all participants before data and biological specimen collection. All the authors have read and approved the paper for publication.

## Supplementary Information

Supplementary Information.

## Data Availability

The datasets used and/or analyzed during the current study are not publicly available due to privacy/ethical restrictions but may be available from the corresponding author on reasonable request.

## Abbreviations

CES-D: Center for epidemiologic studies-depression scale
MMSE: Mini-mental status examination
OR: Odds ratio
CI: Confidence interval
