# Supplementary material for: Sleep habits are associated with cognition decline in physically robust, but not in frail participants: a longitudinal observational study
Source: Sci Rep. 2022 Jul 8;12:11595. doi: 10.1038/s41598-022-15915-y (PMC9270465; doi:10.1038/s41598-022-15915-y)
Supplement: Supplementary file 1 — Supplementary Information. [file 41598_2022_15915_MOESM1_ESM.docx]

**Sleep habits are associated with cognition decline in physically robust, but not in frail participants: a longitudinal observational study**

Shu-Chun Chuang^a,*^, I-Chien Wu^a^, Jen-Jen Chang^b^, Yi-Fen Tsai^a^, Chiu-Wen Cheng^a^, Yen-Feng Chiu^a^, Hsing-Yi Chang^a^, Marion M. Lee^c^, Chih-Cheng Hsu^a^, Chao Agnes Hsiung^a^

1. Institute of Population Health Sciences, National Health Research Institutes, Zhunan, Miaoli, Taiwan
2. Department of Epidemiology and Biostatistics, College for Public Health & Social Justice, Saint Louis University, St. Louis, MO, USA
3. Department of Epidemiology and Biostatistics, University of California San Francisco, San Francisco, CA, USA

**Corresponding author:**

Shu-Chun Chuang, Ph.D.

Institute of Population Health Sciences

National Health Research Institutes

Zhunan, Miaoli, Taiwan

Phone: +886 37 246 166 ext. 36314

Fax: +886 37 586 467

Email: [scchuang@nhri.org.tw](mailto:scchuang@nhri.org.tw)

Supplementary Table 1. Baseline characteristics for participants included and not included in the study. (A) compare those who have finished the follow-up assessments and those who have not yet scheduled the assessments at the time of the analyses (B) compare those who participated in the follow-up and those who have died or dropped from the study among the participants who were scheduled to finish the follow-up assessments^1^.

|  | A | | | | | B | | | | | | |
| --- | --- | --- | --- | --- | --- | --- | --- | --- | --- | --- | --- | --- |
| Characteristics at baseline | Finished | | Not yet scheduled | |  | Completed | | Death or too ill  to participate | | Refuse or  withdraw | |  |
|  | N | % | N | % | *p*-value^3^ | N | % | N | % | N | % | *p*-value^4^ |
| **Age (mean ± SD)** | 69.7±8.2 | | 69.4±8.3 | | 0.128 | 68.3±7.7 | | 76.4±7.6 | | 70.3±8.2 | | <0.001 |
| **Sex** |  |  |  |  |  |  |  |  |  |  |  |  |
| Men | 1466 | 47.1 | 1205 | 47.5 | 0.773 | 1030 | 47.3 | 252 | 59.6 | 184 | 35.9 | <0.001 |
| Women | 1647 | 52.9 | 1333 | 52.5 |  | 1147 | 52.7 | 171 | 40.4 | 329 | 64.1 |  |
| **Education** |  |  |  |  |  |  |  |  |  |  |  |  |
| Illiteracy | 439 | 14.1 | 179 | 7.1 | <0.001 | 244 | 11.2 | 97 | 23.0 | 98 | 19.1 | <0.001 |
| Primary school | 1484 | 47.7 | 1012 | 39.9 |  | 1013 | 46.5 | 203 | 48.1 | 268 | 52.2 |  |
| More than primary school | 1189 | 38.2 | 1343 | 53.0 |  | 920 | 42.3 | 122 | 28.9 | 147 | 28.7 |  |
| **Current working status** |  |  |  |  |  |  | |  |  |  |  |  |
| No | 2289 | 73.5 | 1929 | 76.0 | 0.034 | 1536 | 70.6 | 364 | 86.1 | 389 | 75.8 | <0.001 |
| Yes | 824 | 26.5 | 609 | 24.0 |  | 641 | 29.4 | 59 | 13.9 | 124 | 24.2 |  |
| **Smoking** |  |  |  |  |  |  |  |  |  |  |  |  |
| Never | 2187 | 70.3 | 1803 | 71.0 | 0.330 | 1562 | 71.8 | 243 | 57.5 | 382 | 74.5 | <0.001 |
| Former | 510 | 16.4 | 429 | 16.9 |  | 345 | 15.8 | 105 | 24.8 | 60 | 11.7 |  |
| Current | 416 | 13.4 | 306 | 12.1 |  | 270 | 12.4 | 75 | 17.7 | 71 | 13.8 |  |
| **Drinking** |  |  |  |  |  |  |  |  |  |  |  |  |
| Never | 1994 | 64.1 | 1442 | 56.8 | <0.001 | 1357 | 62.3 | 277 | 65.6 | 360 | 70.2 | <0.001 |
| Former | 294 | 9.4 | 297 | 11.7 |  | 187 | 8.6 | 70 | 16.5 | 37 | 7.2 |  |
| Current | 825 | 26.5 | 799 | 31.5 |  | 633 | 29.1 | 76 | 18.0 | 116 | 22.6 |  |
| **Betel chewing** |  |  |  |  |  |  |  |  |  |  |  |  |
| Never | 2766 | 88.9 | 2193 | 86.4 | 0.013 | 1934 | 88.8 | 370 | 87.5 | 462 | 90.1 | 0.578 |
| Former | 262 | 8.4 | 250 | 9.9 |  | 180 | 8.3 | 43 | 10.2 | 39 | 7.6 |  |
| Current | 85 | 2.7 | 95 | 3.7 |  | 63 | 2.9 | 10 | 2.4 | 12 | 2.3 |  |
| **Exercise** |  |  |  |  |  |  |  |  |  |  |  |  |
| No | 509 | 16.4 | 522 | 20.6 | <0.001 | 320 | 14.7 | 97 | 22.9 | 92 | 18.0 | <0.001 |
| Some | 2171 | 69.8 | 1811 | 71.4 |  | 1528 | 70.2 | 286 | 67.6 | 357 | 69.7 |  |
| Meet the recommendation^2^ | 432 | 13.9 | 204 | 8.0 |  | 329 | 15.1 | 40 | 9.5 | 63 | 12.3 |  |
| **Number of Chronic Diseases** |  |  |  |  |  |  | |  |  |  |  |  |
| 0-2 | 1306 | 42.0 | 1022 | 40.3 | 0.122 | 969 | 44.5 | 134 | 31.7 | 203 | 39.6 | <0.001 |
| 3-5 | 1382 | 44.4 | 1123 | 44.3 |  | 936 | 43.0 | 196 | 46.3 | 250 | 48.7 |  |
| ≥6 | 425 | 13.7 | 393 | 15.5 |  | 272 | 12.5 | 93 | 22.0 | 60 | 11.7 |  |
| **Social network** |  |  |  |  |  |  |  |  |  |  |  |  |
| ≥8 | 1675 | 53.8 | 1065 | 42.0 | <0.001 | 1231 | 56.6 | 178 | 42.1 | 266 | 51.9 | <0.001 |
| 6-7 | 830 | 16.7 | 746 | 29.4 |  | 577 | 26.5 | 115 | 27.2 | 138 | 26.9 |  |
| 0-5 | 608 | 19.5 | 727 | 28.6 |  | 369 | 17.0 | 130 | 30.7 | 109 | 21.2 |  |
| **CESD** |  |  |  |  |  |  |  |  |  |  |  |  |
| <16 | 2943 | 94.5 | 2391 | 94.3 | 0.676 | 2075 | 95.3 | 390 | 92.2 | 478 | 93.2 | 0.011 |
| ≥16 | 170 | 5.5 | 145 | 5.7 |  | 102 | 4.7 | 33 | 7.8 | 35 | 6.8 |  |
|  |  |  |  |  |  |  |  |  |  |  |  |  |
| MMSE (mean±SD) | 25.92±3.95 | | 26.40±3.52 | | 0.001 | 26.53±3.58 | | 23.64±4.57 | | 25.20±4.12 | | <0.001 |

1. Participants who self-reported being diagnosed with dementia by a physician at baseline were excluded.
2. Moderate exercise or work-related physical activity 150 min/week or vigorous exercise or work-related physical activity 75 min/week
3. Compared between the “finished” and “not yet scheduled” groups.
4. Compared among the "completed”, “death or too ill to participate”, and “refuse or withdraw” groups.

Supplementary Table 2. Sensitivity analysis of association between sleep parameters and substantial decline in MMSE score. A) MMSE decline by ≥4 points and B) among whose baseline MMSE score ≥24.

|  | Total | | | | | | Robust | | | | | | Prefrail | | | | | | Frail | | | | | |
| --- | --- | --- | --- | --- | --- | --- | --- | --- | --- | --- | --- | --- | --- | --- | --- | --- | --- | --- | --- | --- | --- | --- | --- | --- |
|  | A | | | B^1^ | | | A | | | B^1^ | | | A | | | B^1^ | | | A | | | B^1^ | | |
|  | OR^2^ | 95% CI | | OR^2^ | 95% CI | | OR^2^ | 95% CI | | OR^2^ | 95% CI | | OR^2^ | 95% CI | | OR^2^ | 95% CI | | OR^2^ | 95% CI | | OR^2^ | 95% CI | |
| **N** (declined / non-declined) | 423 / 1709 | | | 537 / 1311 | | | 194 / 1033 | | | 294 / 839 | | | 193 / 593 | | | 217 / 428 | | | 25 / 45 | | | 18 / 20 | | |
| **Midpoint of sleep** | | | |  |  |  |  | | |  |  |  |  | | |  |  |  |  |  |  |  |  |  |
| 23:00-01:00 | 1.04 | (0.77, | 1.41) | 0.93 | (0.65, | 1.32) | 1.22 | (0.80, | 1.84) | 1.05 | (0.66, | 1.66) | 0.86 | (0.53, | 1.36) | 0.65 | (0.36, | 1.16) | 0.82 | (0.23, | 2.82) | NE^5^ | | |
| 01:00-03:00 | 1.00 |  |  | 1.00 |  |  | 1.00 |  |  | 1.00 |  |  | 1.00 |  |  | 1.00 |  |  | 1.00 |  |  |  |  |  |
| 03:00-05:00 | 0.86 | (0.64, | 1.17) | 0.88 | (0.65, | 1.21) | 0.78 | (0.51, | 1.15) | 0.74 | (0.48, | 1.11) | 1.15 | (0.71, | 1.85) | 1.30 | (0.78, | 2.15) | 0.27 | (0.03, | 1.49) |  |  |  |
| 05:00-23:00 | 0.67 | (0.31, | 1.45) | 0.68 | (0.31, | 1.50) | 0.77 | (0.23, | 2.10) | 0.76 | (0.23, | 2.13) | 1.08 | (0.35, | 2.90) | 1.07 | (0.34, | 2.96) | NE^6^ | | |  |  |  |
| **Total Sleep duration (h)** | | | |  |  |  |  | | |  |  |  |  | | |  |  |  |  |  |  |  |  |  |
| <5 | 1.41 | (1.00, | 1.98) | 1.57 | (1.06, | 2.33) | 1.86 | (1.17, | 2.95) | 2.18 | (1.31, | 3.60) | 1.02 | (0.60, | 1.74) | 0.98 | (0.51, | 1.86) | 1.05 | (0.25, | 4.40) | 1.12 | (0.16, | 7.87) |
| 5-7 | 1.01 | (0.81, | 1.26) | 0.99 | (0.78, | 1.26) | 1.06 | (0.79, | 1.42) | 1.06 | (0.78, | 1.44) | 1.00 | (0.69, | 1.44 | 0.94 | (0.62, | 1.41) | 0.52 | (0.13, | 2.03) | 0.66 | (0.08, | 4.89) |
| 7-9 | 1.00 |  |  | 1.00 |  |  | 1.00 |  |  | 1.00 |  |  | 1.00 |  |  | 1.00 |  |  | 1.00 |  |  | 1.00 |  |  |
| ≥9 | 1.28 | (0.87, | 1.88) | 1.31 | (0.86, | 2.00) | 1.45 | (0.83, | 2.49) | 1.60 | (0.90, | 2.82) | 1.06 | (0.59, | 1.90) | 1.07 | (0.55, | 2.05) | 1.40 | (0.34, | 5.95) | 1.10 | (0.17, | 6.82) |
| **Nap** |  |  |  |  |  |  |  |  |  |  |  |  |  |  |  |  |  |  |  |  |  |  |  |  |
| No | 1.00 |  |  | 1.00 |  |  | 1.00 |  |  | 1.00 |  |  | 1.00 |  |  | 1.00 |  |  | 1.00 |  |  | 1.00 |  |  |
| Yes | 1.13 | (0.92, | 1.38) | 1.18 | (0.94, | 1.48) | 1.13 | (0.86, | 1.48) | 1.17 | (0.88, | 1.55) | 1.15 | (0.83, | 1.59) | 1.32 | (0.91, | 1.93) | 1.04 | (0.38, | 2.77) | 0.34 | (0.06, | 1.51) |
| **Excessive daytime sleepiness** | | | |  |  |  |  | | |  |  |  |  | | |  |  |  |  |  |  |  |  |  |
| ≤10 | 1.00 |  |  | 1.00 |  |  | 1.00 |  |  | 1.00 |  |  | 1.00 |  |  | 1.00 |  |  | 1.00 |  |  | 1.00 |  |  |
| >10 | 1.26 | (0.95, | 1.66) | 1.15 | (0.85, | 1.55) | 1.48 | (1.03, | 2.11) | 1.47 | (1.01, | 2.11) | 1.01 | (0.62, | 1.61) | 0.80 | (0.46, | 1.36) | 1.46 | (0.43, | 5.07) | 0.94 | (0.17, | 4.82) |
| **Sleep efficiency** | | | |  |  |  |  | | |  |  |  |  | | |  |  |  |  |  |  |  |  |  |
| ≥85% | 1.00 |  |  | 1.00 |  |  | 1.00 |  |  | 1.00 |  |  | 1.00 |  |  | 1.00 |  |  | 1.00 |  |  | 1.00 |  |  |
| 75-85% | 0.90 | (0.69, | 1.17) | 0.92 | (0.70, | 1.22) | 0.85 | (0.60, | 1.19) | 0.79 | (0.54, | 1.13) | 0.95 | (0.62, | 1.45) | 1.15 | (0.72, | 1.83) | 0.66 | (0.14, | 2.94) | 1.97 | (0.21, | 22.6) |
| 65-75% | 0.94 | (0.67, | 1.30) | 1.04 | (0.73, | 1.49) | 1.02 | (0.66, | 1.57) | 1.08 | (0.68, | 1.68) | 0.82 | (0.48, | 1.37) | 0.96 | (0.52, | 1.72) | 0.84 | (0.18, | 3.92) | 3.86 | (0.41, | 67.6) |
| <65% | 1.21 | (0.90, | 1.63) | 1.31 | (0.93, | 1.83) | 1.62 | (1.07, | 2.43) | 1.59 | (1.01, | 2.47) | 0.97 | (0.61, | 1.51) | 1.09 | (0.63, | 1.87) | 0.65 | (0.15, | 2.58) | 1.45 | (0.15, | 16.3) |
| **Insomnia Symptom**^3^ | | | |  |  |  |  | | |  |  |  |  | | |  |  |  |  |  |  |  |  |  |
| No | 1.00 |  |  | 1.00 |  |  | 1.00 |  |  | 1.00 |  |  | 1.00 |  |  | 1.00 |  |  | 1.00 |  |  | 1.00 |  |  |
| Yes | 1.38 | (0.88, | 2.18) | 1.56 | (0.93, | 2.61) | 2.31 | (1.22, | 4.32) | 2.75 | (1.37, | 5.44) ^6^ | 0.71 | (0.34, | 1.41) | 0.73 | (0.31, | 1.62) | 2.76 | (0.56, | 15.4) | 3.35 | (0.36, | 47.9) |
| **Insomnia diagnosis** | | | |  |  |  |  | | |  |  |  |  | | |  |  |  |  |  |  |  |  |  |
| No | 1.00 |  |  | 1.00 |  |  | 1.00 |  |  | 1.00 |  |  | 1.00 |  |  | 1.00 |  |  | 1.00 |  |  | 1.00 |  |  |
| Yes | 0.94 | (0.64, | 1.37) | 0.96 | (0.64, | 1.43) | 1.52 | (0.89, | 2.54) | 1.62 | (0.93, | 2.77) | 0.55 | (0.30, | 0.97) | 0.53 | (0.28, | 0.96) | 1.12 | (0.22, | 5.95) | 2.12 | (0.22, | 27.2) |
| **Self-Reported hypnotic or sedative drug use** | | | |  | | |  | | |  |  |  |  | | |  |  |  |  |  |  |  |  |  |
| No | 1.00 |  |  | 1.00 |  |  | 1.00 |  |  | 1.00 |  |  | 1.00 |  |  | 1.00 |  |  | 1.00 |  |  | 1.00 |  |  |
| Yes | 0.78 | (0.58, | 1.06) | 0.79 | (0.57, | 1.09) | 0.82 | (0.53, | 1.25) | 0.88 | (0.56, | 1.35) | 0.72 | (0.45, | 1.12) | 0.66 | (0.40, | 1.07) | 0.99 | (0.27, | 3.57) | 1.49 | (0.22, | 14.7) |
| **Sleep summary score**^4^ | | | |  |  |  |  |  |  |  |  |  |  |  |  |  |  |  |  |  |  |  |  |  |
| 0 | 1.00 |  |  | 1.00 |  |  | 1.00 |  |  | 1.00 |  |  | 1.00 |  |  | 1.00 |  |  | 1.00 |  |  | 1.00 |  |  |
| 1 | 1.10 | (0.86, | 1.40) | 1.07 | (0.83, | 1.39) | 1.30 | (0.95, | 1.78) | 1.33 | (0.96, | 1.84) | 0.87 | (0.59, | 1.28) | 0.79 | (0.51, | 1.21) | 1.31 | (0.43, | 4.16) | 0.77 | (0.15, | 4.71) |
| ≥2 | 2.54 | (1.64, | 3.95)^6^ | 2.70 | (1.66, | 4.38) ^6^ | 3.68 | (2.06, | 6.61) ^6^ | 4.03 | (2.17, | 7.52) ^6^ | 1.37 | (0.65, | 2.87) | 1.39 | (0.58, | 3.30) | 4.43 | (0.73, | 33.6 | 2.94 | (0.37, | 37.2) |

1. Substantial decline was defined as MMSE decline by ≥3 points
2. Odds ratios were adjusted for age at baseline, sex, center, education, current working status, betel nut chewing status, number of chronic diseases, scores for social networking, and CESD (and frailty status).
3. Insomnia was defined as having all symptoms: difficulty in falling asleep, difficulty in staying asleep, and waking up too early.
4. Sleep summary score was created for having short or long sleep duration (<5 or ≥9 hrs), excessive daytime sleepiness, and insomnia symptom.
5. NE: not estimable.
6. *P*-value <0.005

Supplementary Table 3. Associations between sleep and different outcomes: MMSE decline, death or unable to answer, and refuse or withdraw, by nominal logistic regression.

|  | Total | | | | | | | | | Robust | | | | | | | | | Prefrail | | | | | | | | | Frail | | | | | | | | |
| --- | --- | --- | --- | --- | --- | --- | --- | --- | --- | --- | --- | --- | --- | --- | --- | --- | --- | --- | --- | --- | --- | --- | --- | --- | --- | --- | --- | --- | --- | --- | --- | --- | --- | --- | --- | --- |
|  | Diff≤-3 | | | Dead or too ill  to participate | | | Refuse or withdraw | | | Diff≤-3 | | | Dead or too ill  to participate | | | Refuse or withdraw | | | Diff≤-3 | | | Dead or too ill  to participate | | | Refuse or withdraw | | | Diff≤-3 | | | Dead or too ill  to participate | | | Refuse or withdraw | | |
|  | OR | 95% CI | | OR | 95% CI | | OR | 95% CI | | OR | 95% CI | | OR | 95% CI | | OR | 95% CI | | OR | 95% CI | | OR | 95% CI | | OR | 95% CI | | OR | 95% CI | | OR | 95% CI | | OR | 95% CI | |
| **Midpoint of sleep** | | | |  | | |  | | |  | | |  | | |  | | |  | | |  | | |  | |  |  |  |  |  |  |  |  |  |  |
| 23:00-01:00 | 1.07 | (0.80, | 1.44) | 1.67 | (1.19, | 2.34) | 1.13 | (0.79, | 1.62) | 1.20 | (0.79, | 1.82) | 2.56 | (1.46, | 4.47) | 1.20 | (0.72, | 1.99) | 0.93 | (0.59, | 1.47) | 1.46 | (0.92, | 2.31) | 1.08 | (0.63, | 1.85) | 0.87 | (0.22, | 3.37) | 0.67 | (0.16, | 2.80) | 0.63 | (0.09, | 4.30) |
| 01:00-03:00 | 1.00 |  |  | 1.00 |  |  | 1.00 |  |  | 1.00 |  |  | 1.00 |  |  | 1.00 |  |  | 1.00 |  |  | 1.00 |  |  | 1.00 |  |  | 1.00 |  |  | 1.00 |  |  | 1.00 |  |  |
| 03:00-05:00 | 0.85 | (0.63, | 1.15) | 0.97 | (0.64, | 1.48) | 1.16 | (0.82, | 1.64) | 0.77 | (0.51, | 1.16) | 0.88 | (0.41, | 1.91) | 1.15 | (0.72, | 1.83) | 1.12 | (0.70, | 1.79) | 1.07 | (0.60, | 1.91) | 1.06 | (0.59, | 1.91) | 0.16 | (0.02, | 1.09) | 0.52 | (0.10, | 2.63) | 1.48 | (0.26, | 8.50) |
| 05:00-23:00 | 0.65 | (0.30, | 1.39) | 0.67 | (0.23, | 1.94) | 1.89 | (0.97, | 3.70) | 0.71 | (0.23, | 2.19) | 0.89 | (0.11, | 7.51) | 1.40 | (0.45, | 4.36) | 0.89 | (0.31, | 2.58) | 1.03 | (0.29, | 3.69) | 2.48 | (0.98, | 6.23) | NE | | | | | | 0.86 | (0.05, | 16.3) |
| **Total Sleep duration (h)** | | | |  | | |  | | |  | | |  | | |  | | |  | | |  | | |  | | |  |  |  |  |  |  |  |  |  |
| <5 | 1.42 | (1.02, | 1.98) | 1.08 | (0.69, | 1.69) | 0.97 | (0.63, | 1.48) | 1.86 | (1.16, | 2.96) | 1.53 | (0.69, | 3.42) | 0.92 | (0.49, | 1.72) | 1.09 | (0.65, | 1.83) | 1.11 | (0.62, | 2.00) | 0.84 | (0.44, | 1.62) | 1.03 | (0.21, | 5.03) | 0.30 | (0.04, | 2.14) | 1.32 | (0.20, | 8.53) |
| 5-7 | 1.04 | (0.83, | 1.29) | 0.94 | (0.70, | 1.27) | 1.03 | (0.79, | 1.34) | 1.07 | (0.80, | 1.43) | 0.98 | (0.60, | 1.60) | 1.15 | (0.82, | 1.62) | 1.06 | (0.74, | 1.52) | 0.90 | (0.59, | 1.38) | 0.92 | (0.60, | 1.42) | 0.51 | (0.12, | 2.20) | 0.78 | (0.19, | 3.11) | 0.72 | (0.12, | 4.32) |
| 7-9 | 1.00 |  |  | 1.00 |  |  | 1.00 |  |  | 1.00 |  |  | 1.00 |  |  | 1.00 |  |  | 1.00 |  |  | 1.00 |  |  | 1.00 |  |  | 1.00 |  |  | 1.00 |  |  | 1.00 |  |  |
| ≥9 | 1.30 | (0.89, | 1.89) | 1.21 | (0.78, | 1.87) | 1.37 | (0.87, | 2.15) | 1.48 | (0.85, | 2.56) | 1.04 | (0.43, | 2.52) | 1.30 | (0.64, | 2.60) | 1.11 | (0.62, | 1.99) | 1.35 | (0.75, | 2.44) | 1.55 | (0.81, | 2.95) | 1.24 | (0.27, | 5.71) | 0.71 | (0.15, | 3.31) | 0.50 | (0.06, | 3.96) |
| **Nap** |  |  |  |  |  |  |  |  |  |  |  |  |  |  |  |  |  |  |  |  |  |  |  |  |  |  |  |  |  |  |  |  |  |  |  |  |
| No | 1.00 |  |  | 1.00 |  |  | 1.00 |  |  | 1.00 |  |  | 1.00 |  |  | 1.00 |  |  | 1.00 |  |  | 1.00 |  |  | 1.00 |  |  | 1.00 |  |  | 1.00 |  |  | 1.00 |  |  |
| Yes | 1.13 | (0.92, | 1.38) | 0.88 | (0.67, | 1.15) | 0.98 | (0.77, | 1.25) | 1.14 | (0.87, | 1.50) | 1.03 | (0.65, | 1.64) | 1.04 | (0.75, | 1.44) | 1.10 | (0.80, | 1.52) | 0.70 | (0.49, | 1.01) | 0.95 | (0.64, | 1.40) | 1.25 | (0.43, | 3.66) | 1.43 | (0.47, | 4.37) | 0.36 | (0.09, | 1.39) |
| **Excessive daytime sleepiness** | | | |  | | |  | | |  | | |  | | |  | | |  | | |  | | |  | | |  |  |  |  |  |  |  |  |  |
| ≤10 | 1.00 |  |  | 1.00 |  |  | 1.00 |  |  | 1.00 |  |  | 1.00 |  |  | 1.00 |  |  | 1.00 |  |  | 1.00 |  |  | 1.00 |  |  | 1.00 |  |  | 1.00 |  |  | 1.00 |  |  |
| >10 | 1.26 | (0.96, | 1.66) | 1.01 | (0.70, | 1.45) | 1.16 | (0.83, | 1.61) | 1.52 | (1.07, | 2.17) | 1.10 | (0.58, | 2.09) | 1.02 | (0.64, | 1.62) | 0.95 | (0.60, | 1.51) | 0.88 | (0.53, | 1.48) | 1.17 | (0.68, | 1.99) | 1.46 | (0.39, | 5.50) | 1.05 | (0.28, | 3.89) | 1.69 | (0.34, | 8.34) |
| **Sleep efficiency** | | | |  | | |  | | |  | | |  | | |  | | |  | | |  | | |  |  |  |  |  |  |  |  |  |  |  |  |
| ≥85% | 1.00 |  |  | 1.00 |  |  | 1.00 |  |  | 1.00 |  |  | 1.00 |  |  | 1.00 |  |  | 1.00 |  |  | 1.00 |  |  | 1.00 |  |  | 1.00 |  |  | 1.00 |  |  | 1.00 |  |  |
| 75-85% | 0.89 | (0.69, | 1.15) | 0.92 | (0.65, | 1.30) | 0.87 | (0.64, | 1.18) | 0.84 | (0.59, | 1.19) | 0.94 | (0.52, | 1.70) | 0.68 | (0.44, | 1.04) | 0.95 | (0.62, | 1.43) | 0.92 | (0.56, | 1.51) | 1.24 | (0.76, | 2.00) | 0.49 | (0.11, | 2.20) | 0.50 | (0.12, | 2.15) | 0.37 | (0.06, | 2.42) |
| 65-75% | 0.95 | (0.69, | 1.31) | 1.14 | (0.75, | 1.73) | 0.69 | (0.46, | 1.05) | 1.04 | (0.67, | 1.61) | 1.33 | (0.67, | 2.65) | 0.65 | (0.36, | 1.15) | 0.80 | (0.48, | 1.34) | 1.15 | (0.65, | 2.05) | 0.74 | (0.39, | 1.41) | 0.89 | (0.18, | 4.54) | 0.57 | (0.10, | 3.36) | 0.55 | (0.07, | 4.71) |
| <65% | 1.22 | (0.92, | 1.64) | 1.28 | (0.89, | 1.84) | 1.23 | (0.88, | 1.72) | 1.58 | (1.05, | 2.40) | 1.55 | (0.78, | 3.09) | 1.41 | (0.88, | 2.25) | 1.00 | (0.64, | 1.54) | 1.24 | (0.76, | 2.01) | 1.16 | (0.69, | 1.94) | 0.58 | (0.15, | 2.28) | 0.63 | (0.16, | 2.53) | 0.55 | (0.11, | 2.79) |
| **Insomnia Symptom** | | | |  | | |  | | |  | | |  | | |  | | |  | | |  | | |  | |  |  |  |  |  |  |  |  |  |  |
| No | 1.00 |  |  | 1.00 |  |  | 1.00 |  |  | 1.00 |  |  | 1.00 |  |  | 1.00 |  |  | 1.00 |  |  | 1.00 |  |  | 1.00 |  |  | 1.00 |  |  | 1.00 |  |  |  |  |  |
| Yes | 1.36 | (0.87, | 2.11) | 0.86 | (0.49, | 1.61) | 1.28 | (0.77, | 2.14) | 2.16 | (1.15, | 4.05) | 1.07 | (0.30, | 3.79) | 1.83 | (0.88, | 3.78) | 0.79 | (0.39, | 1.58) | 0.81 | (0.38, | 1.72) | 0.61 | (0.25, | 1.47) | 3.45 | (0.57, | 20.9) | 1.26 | (0.17, | 9.13) | 10.4 | (1.39, | 77.1) |
| **Insomnia Diagnosis** | | | |  | | |  | | |  | | |  | | |  | | |  | | |  | | |  | |  |  |  |  |  |  |  |  |  |  |
| No | 1.00 |  |  | 1.00 |  |  | 1.00 |  |  | 1.00 |  |  | 1.00 |  |  | 1.00 |  |  | 1.00 |  |  | 1.00 |  |  | 1.00 |  |  | 1.00 |  |  | 1.00 |  |  | 1.00 |  |  |
| Yes | 1.56 | (0.92, | 2.63) | 1.14 | (0.44, | 2.96) | 1.18 | (0.61, | 2.27) | 0.57 | (0.32, | 1.02) | 1.09 | (0.62, | 1.93) | 0.68 | (0.36, | 1.30) | 1.32 | (0.24, | 7.28) | 1.50 | (0.30, | 7.40) | 0.82 | (0.09, | 7.18) | 1.56 | (0.92, | 2.63) | 1.14 | (0.44, | 2.96) | 1.18 | (0.61, | 2.27) |
| **Hypnotic or sedative drug use** | | | |  | | |  | | |  | | |  | | |  | | |  | | |  | | |  | | |  |  |  |  |  |  |  |  |  |
| No | 1.00 |  |  | 1.00 |  |  | 1.00 |  |  | 1.00 |  |  | 1.00 |  |  | 1.00 |  |  | 1.00 |  |  | 1.00 |  |  | 1.00 |  |  | 1.00 |  |  | 1.00 |  |  | 1.00 |  |  |
| Yes | 0.79 | (0.59, | 1.05) | 1.14 | (0.81, | 1.60) | 1.07 | (0.77, | 1.48) | 0.82 | (0.54, | 1.25) | 0.80 | (0.40, | 1.59) | 1.02 | (0.64, | 1.63) | 0.72 | (0.46, | 1.12) | 1.24 | (0.79, | 1.94) | 1.22 | (0.76, | 1.97) | 0.96 | (0.28, | 3.29) | 1.63 | (0.48, | 5.55) | 0.42 | (0.07, | 2.54) |
| **Sleep summary score** | | | |  |  |  |  |  |  |  |  |  |  |  |  |  |  |  |  |  |  |  |  |  |  |  |  |  |  |  |  |  |  |  |  |  |
| 0 | 1.00 |  |  | 1.00 |  |  | 1.00 |  |  | 1.00 |  |  | 1.00 |  |  | 1.00 |  |  | 1.00 |  |  | 1.00 |  |  | 1.00 |  |  | 1.00 |  |  |  |  |  |  |  |  |
| 1 | 1.12 | (0.89, | 1.42) | 1.05 | (0.78, | 1.42) | 1.17 | (0.88, | 1.55) | 1.32 | (0.96, | 1.81) | 1.11 | (0.65, | 1.89) | 1.07 | (0.72, | 1.57) | 0.89 | (0.61, | 1.31) | 1.06 | (0.70, | 1.61) | 1.24 | (0.80, | 1.92) | 1.16 | (0.35, | 3.84) | 0.55 | (0.17, | 1.76) | 0.71 | (0.14, | 3.50) |
| ≥2 | 2.36 | (1.53, | 3.62)^1^ | 1.45 | (0.82, | 2.55) | 1.41 | (0.80, | 2.47) | 3.64 | (2.03, | 6.52) ^1^ | 1.25 | (0.39, | 4.03) | 1.46 | (0.65, | 3.27) | 1.26 | (0.61, | 2.57) | 1.38 | (0.64, | 2.98) | 0.97 | (0.39, | 2.42) | 4.36 | (0.57, | 33.3) | 1.28 | (0.17, | 9.49) | 3.11 | (0.30, | 32.2) |

1. *P*-value <0.005

Odds ratios were adjusted for age at baseline, sex, center, education, current working status, betel nut chewing status, number of chronic diseases, scores for social networking, and CESD (and frailty status for the total). Summary score was created for having short or long sleep duration (<5 or ≥9 hrs), excessive daytime sleepiness, and insomnia symptom.

NE: not estimable.

Supplementary Table 4. Association between baseline sleep parameters and MMSE decline^1^ combining prefrail and frail participants

|  | Prefrail+Frail | | | | | P for  heterogeneity |
| --- | --- | --- | --- | --- | --- | --- |
|  | Diff>-3 | Diff≤-3 | OR^2^ | 95% CI | |  |
| **Midpoint of sleep** | |  |  |  |  |  |
| 23:00-01:00 | 70 | 49 | 0.86 | (0.55, | 1.34) | 0.834 |
| 01:00-03:00 | 366 | 209 | 1.00 |  |  |  |
| 03:00-05:00 | 93 | 37 | 1.01 | (0.64, | 1.62) |  |
| 05:00-23:00 | 22 | 5 | 0.69 | (0.24, | 2.01) |  |
| **Total sleep duration (h)** | | |  |  |  |  |
| <5 | 69 | 42 | 1.07 | (0.65, | 1.77) | 0.452 |
| 5-7 | 227 | 112 | 0.95 | (0.67, | 1.35) |  |
| 7-9 | 204 | 110 | 1.00 |  |  |  |
| ≥9 | 48 | 35 | 1.18 | (0.69, | 2.03) |  |
| **Nap** |  |  |  |  |  |  |
| No | 248 | 115 | 1.00 |  |  | 0.816 |
| Yes | 304 | 185 | 1.14 | (0.83, | 1.56) |  |
| **Excessive daytime sleepiness** | | | |  |  |  |
| ≤10 | 421 | 216 | 1.00 |  |  | 0.311 |
| >10 | 82 | 45 | 1.06 | (0.68, | 1.66) |  |
| **Sleep efficiency** | |  |  |  |  |  |
| ≥85% | 258 | 144 | 1.00 |  |  | 0.275 |
| 75-85% | 115 | 61 | 0.95 | (0.63, | 1.43) |  |
| 65-75% | 71 | 34 | 0.85 | (0.52, | 1.39) |  |
| <65% | 103 | 60 | 0.95 | (0.62, | 1.45) |  |
| **Insomnia complaint** | |  |  |  |  |  |
| No | 514 | 282 | 1.00 |  |  | 0.016 |
| Yes | 38 | 18 | 0.84 | (0.44, | 1.61) |  |
| **Insomnia diagnosis** | |  |  |  |  |  |
| No | 476 | 277 | 1.00 |  |  | 0.016 |
| Yes | 76 | 23 | 0.61 | (0.35, | 1.05) |  |
| **Self-reported hypnotic or sedative drug use** | | | | | |  |
| No | 439 | 255 | 1.00 |  |  | 0.637 |
| Yes | 113 | 45 | 0.75 | (0.49, | 1.15) |  |
| **Sleep summary score**^3^ |  |  |  |  |  |  |
| 0 | 325 | 163 | 1.00 |  |  | 0.090 |
| 1 | 144 | 76 | 0.85 | (0.56, | 1.30) |  |
| ≥2 | 30 | 21 | 1.65 | (0.75, | 3.65) |  |

1. A substantial MMSE decline was defined as a 3 or more point drop in MMSE score at the follow-up assessment.
2. Odds ratios were adjusted for age at the baseline assessment, sex, center, education, current working status, betel nut chewing status, number of chronic diseases, social network score, and CES-D score (and physical frailty status for total).
3. Sleep summary score was created for having short or long sleep duration (<5 or ≥9 h), excessive daytime sleepiness, and insomnia complaints.
